# An interpretable machine-learning model for early prediction of acute kidney injury in polytrauma patients

**DOI:** 10.3389/fmed.2026.1750815

**Published:** 2026-07-20

**Authors:** Yang He, Xidong Wang, Jiali Huang, Jinglan Liu

**Affiliations:** 1 Department of Critical Care Medicine, The College of Clinical Medical Science, China Three Gorges University, Yichang Central People's Hospital, Yichang, P.R. China; 2 Department of Nursing, Yichang Central People's Hospital, Yichang, P.R. China

**Keywords:** acute kidney injury, eICU-CRD, interpretable machine learning, logistic regression, MIMIC-IV, polytrauma, SHAP

## Abstract

**Objective:**

Acute kidney injury (AKI) is a frequent and clinically important complication after polytrauma, but early risk stratification remains challenging because conventional diagnostic criteria depend on delayed changes in serum creatinine and urine output. This study aimed to develop and interpret an early prediction model for incident AKI in adult ICU patients with polytrauma using routinely available data from the first 6 h after ICU admission.

**Methods:**

We conducted a retrospective observational study using MIMIC-IV as the primary development and internal validation dataset. Incident AKI was defined according to KDIGO criteria and assessed between 7 and 72 h after ICU admission. Candidate predictors measured within the first 6 h were processed using training-set imputation, encoding, and standardization. LASSO regression was used for feature selection. Seven machine-learning algorithms were trained using the retained predictors after applying SMOTE combined with random undersampling in the training set only. Model performance was evaluated using discrimination, precision-recall performance, threshold-based metrics, calibration, Brier score, and decision curve analysis. SHAP was used to interpret the selected primary model. A secondary eICU-CRD analysis assessed cross-database reproducibility.

**Results:**

The MIMIC-IV cohort included 4,287 adult polytrauma ICU patients, of whom 623 developed AKI. LASSO retained 15 early predictors. In the internal test set, logistic regression achieved the highest AUC and AUPRC among the evaluated models (AUC 0.900, 95% CI 0.878–0.923; AUPRC 0.615, 95% CI 0.533–0.703), with high sensitivity (0.888) and negative predictive value (0.974). In the eICU-CRD cohort, SVM showed the highest external AUC (0.709), while logistic regression demonstrated moderate transportability (AUC 0.678). SHAP identified SOFA score, weight, magnesium, antihypertensive medication exposure, age, respiratory rate, heart rate, platelet count, urine output, and temperature as major contributors.

**Conclusion:**

A parsimonious 15-variable logistic regression model using 6-h ICU data showed strong internal performance and clinically interpretable risk signals for early AKI prediction after polytrauma. External validation indicated partial transportability, supporting further prospective validation and local recalibration before clinical implementation.

## 1 Introduction

Trauma remains a leading global cause of death and disability, imposing a substantial socioeconomic burden on healthcare systems (1). In patients with multiple trauma, acute kidney injury (AKI) is among the most frequent and consequential complications, with reported incidence varying by injury severity, critical illness burden, and diagnostic criteria (2–4). AKI is associated with greater organ dysfunction and mortality, making timely recognition of high-risk patients a central challenge in trauma critical care (1, 5, 6).

AKI is defined by an abrupt decline in kidney function that may reflect structural injury, functional impairment, or both (7). In polytrauma, AKI pathogenesis is multifactorial and may involve hypovolemia, systemic inflammation, rhabdomyolysis, hemodynamic instability, and nephrotoxic exposure (8, 9). However, conventional diagnosis still depends largely on delayed changes in serum creatinine and urine output, which narrows the window for preventive or mitigating interventions (10, 11).

Machine-learning approaches can integrate multidimensional clinical data and have shown promise for early AKI prediction in critical care settings (12, 13). Applications include AKI prediction after cardiac surgery, risk stratification in general ICU populations, and continuous inpatient surveillance (14–16). Nevertheless, models developed in heterogeneous ICU cohorts may not perform optimally in polytrauma, where rapid physiologic change, resuscitation effects, tissue injury, and competing organ failures can alter predictor behavior (17, 18). In parallel, interpretable modeling approaches such as SHAP (Shapley Additive exPlanations) can improve transparency by showing how each feature contributes to both global model behavior and individual predictions (19, 20).

Motivated by these gaps, this study aimed to develop a parsimonious and interpretable machine-learning model for early AKI prediction in polytrauma patients using routinely available data from the first 6 h after ICU admission.

## 2 Methods

### 2.1 Data source

We performed a retrospective observational study using two publicly available, de-identified critical care databases: MIMIC-IV and the eICU Collaborative Research Database (eICU-CRD). MIMIC-IV, curated by the MIT Laboratory for Computational Physiology in collaboration with Beth Israel Deaconess Medical Center and Philips Healthcare, contains comprehensive ICU and hospital data for patients treated between 2008 and 2022 (21). eICU-CRD is a multicenter critical care database containing more than 200,000 ICU admissions from multiple U.S. centers between 2014 and 2015 (22). The MIMIC-IV cohort served as the primary dataset for model development and internal validation. eICU-CRD was used as an independent external validation cohort to assess cross-database transportability using the same retained predictor set. Investigators completed the required data-use training (certification ID: 13278787). Because both databases are publicly available and de-identified, additional institutional review board approval was not required.

### 2.2 Study population

Inclusion criteria were: (1) adult patients (≥18 years) with a first ICU admission for trauma; (2) trauma confirmed by any of the following: trauma-related ICD-9/10 diagnosis codes (S00–T79, V00–Y99); and (3) polytrauma or severe trauma defined by either trauma-related diagnosis codes involving two or more distinct anatomical regions or at least one severe injury code (e.g., intracranial hemorrhage, grade ≥ III solid-organ laceration, or major vascular injury). Exclusion criteria were: (1) end-stage renal disease (ESRD); (2) history of kidney transplantation; (3) missing key temporal information precluding ascertainment of hospital or ICU timestamps; (4) lack of data required to ascertain AKI (no in-hospital serum creatinine and no urine output records); (5) hospital length of stay < 24 h, insufficient for AKI ascertainment; and (6) ICU stays that were not the first during the index hospitalization (only the first ICU stay per hospitalization was retained).

AKI was adjudicated according to KDIGO criteria (23), and the outcome window was 7–72 h after ICU admission. Baseline creatinine preferentially used the most recent preadmission value; if unavailable, the lowest creatinine within 48 h after admission was used as a surrogate.

### 2.3 Exposure and covariates

Patients meeting the prespecified eligibility criteria were included and stratified into AKI and non-AKI groups according to KDIGO, for subsequent comparative analyses. All candidate predictors were extracted within the first 6 h of ICU stay to ensure temporal consistency. Based on clinical semantics and data provenance, variables were organized into a structured multidimensional framework covering: demographic baseline characteristics; comorbidities and medication history; widely used severity scores (APACHE, SAPS, and SOFA); dynamic vital signs; systematic laboratory panels (respiratory and acid–base status, inflammatory markers and blood counts, hematology and coagulation, hepatic and renal function, and metabolic parameters); and key treatment and process indicators (e.g., fluid management, organ support, and specific interventions).

### 2.4 Statistical analysis

All analyses were performed in Python v3.10.0. Categorical variables are presented as *n* (%), and continuous variables as median (interquartile range). Between-group comparisons were performed using the chi-square test or Fisher's exact test for categorical variables and the Mann–Whitney *U*-test for continuous variables, as appropriate. All tests were two-sided, and *P* < 0.05 was considered statistically significant. Variables with >20% missingness were excluded.

The MIMIC-IV cohort was randomly divided into training and test sets at an 8:2 ratio using stratified sampling. All preprocessing steps requiring parameter estimation, including imputation, standardization, feature selection, resampling, hyperparameter tuning, and threshold selection, were performed within the training set only. Missing categorical and continuous variables were imputed using the mode and median, respectively. Categorical variables were one-hot encoded, and continuous variables were *z*-score standardized.

Feature selection was performed using L1-penalized logistic regression without univariate prefiltering, with the regularization strength selected by stratified 5-fold cross-validation. The 15 nonzero predictors with the largest absolute coefficients were retained for model development. Class imbalance was addressed by applying SMOTE combined with random undersampling to the training set only.

Seven machine learning algorithms were evaluated using the same selected predictors: logistic regression, decision tree, random forest, XGBoost, support vector machine, artificial neural network, and gradient boosting machine. Hyperparameters were tuned within the training set using nested cross-validation, with an outer 5-fold loop and an inner 3-fold loop. Final models were retrained on the full training set and evaluated in the held-out test set. Decision thresholds were determined in the training set using the Youden index and then applied to the test set.

**Figure 1:**
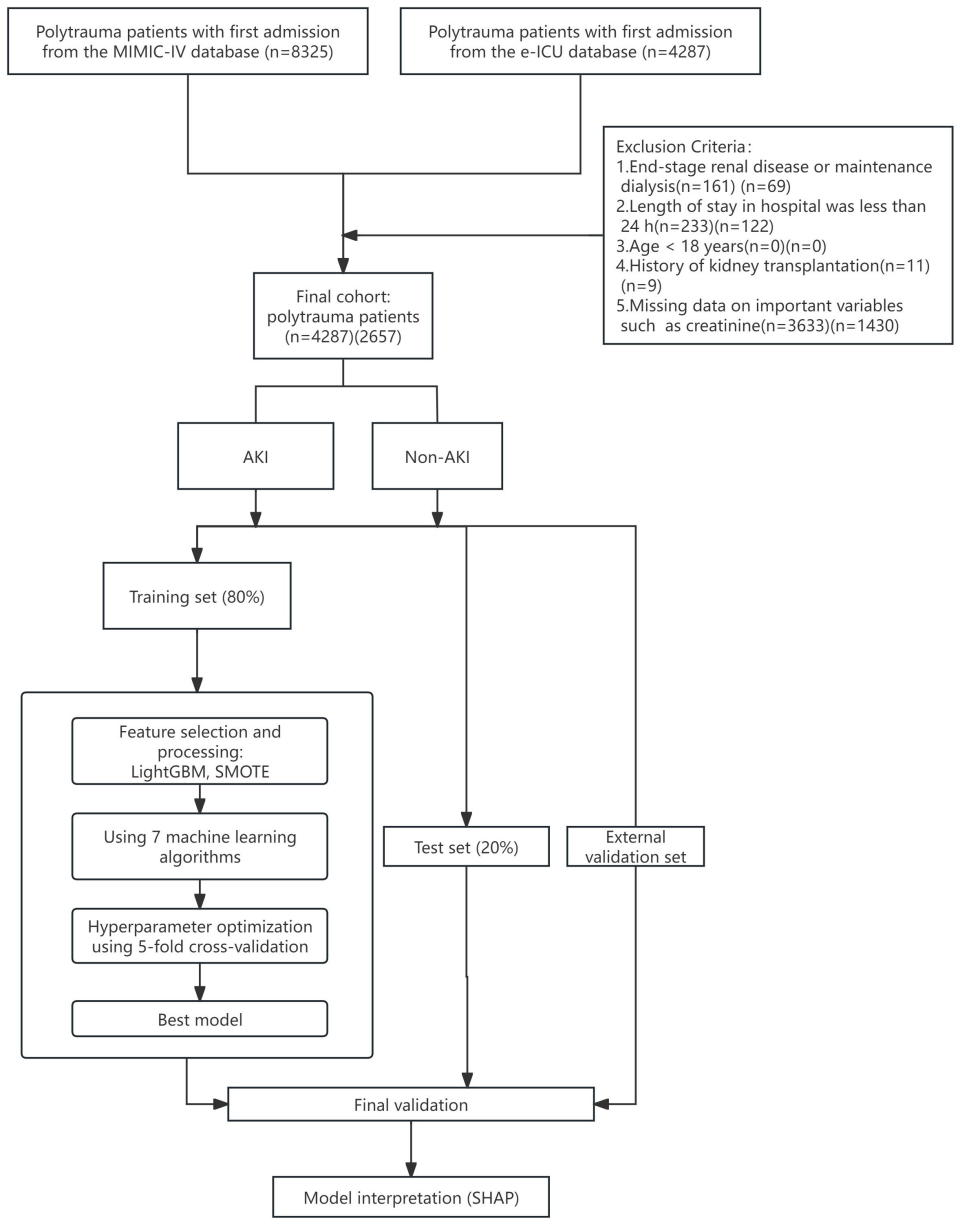
Study flow diagram and model-development workflow.

Model performance was assessed using AUC, AUPRC, sensitivity, specificity, positive predictive value, negative predictive value. Ninety-five percent confidence intervals were estimated using 1,000 bootstrap resamples. Calibration and clinical utility were evaluated using calibration plots and decision curve analysis. The selected primary model was interpreted using SHapley Additive exPlanations (SHAP). For the eICU-CRD cohort, predictor definitions, measurement windows, units, and coding schemes were harmonized with those used in MIMIC-IV whenever possible. The retained 15-variable predictor set was applied to eICU-CRD. A schematic of the full analytical workflow is provided in Figure 1.

## 3 Results

### 3.1 Cohort characteristics

The MIMIC-IV primary cohort included 4,287 adult polytrauma ICU patients. AKI occurred in 623 patients (14.5%) between 7 and 72 h after ICU admission, whereas 3,664 patients (85.5%) did not develop AKI during the prespecified outcome window (Figure 1). Baseline distributions differed across outcome groups across multiple domains, including demographic characteristics, comorbidities, early organ dysfunction, respiratory and hemodynamic measurements, urine output, acid-base status, coagulation-related markers, renal function, and electrolytes. These patterns indicate that the subsequent prediction task incorporated heterogeneous early physiologic and laboratory signals rather than a single dominant clinical dimension (Supplementary Table S1).

### 3.2 Feature selection

**Figure 2:**
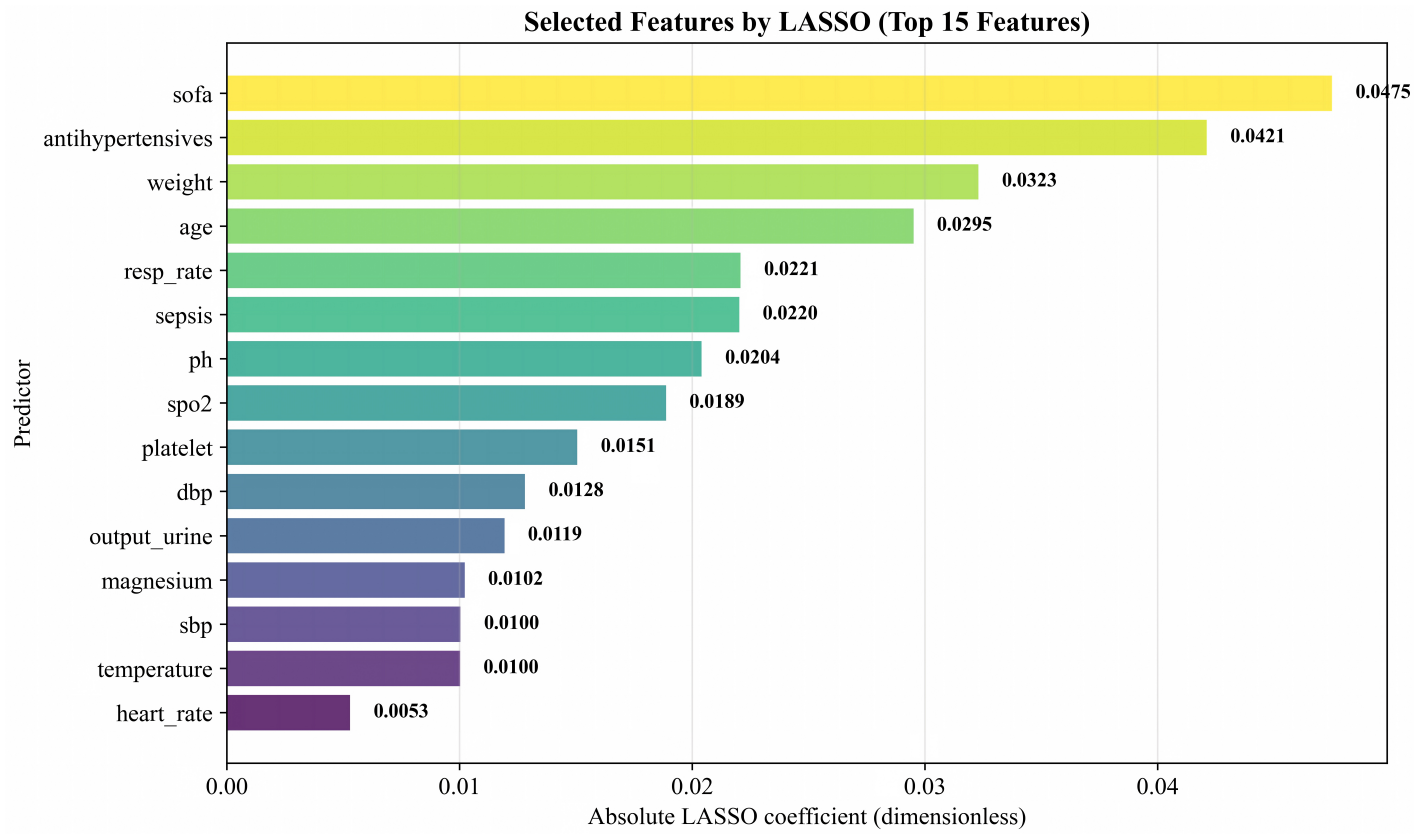
Feature importance ranking of the 15 LASSO-selected predictors. Bars represent absolute LASSO coefficients.

LASSO identified 15 early predictors for subsequent model development (Figure 2; Supplementary Table S2). The largest absolute coefficient was observed for SOFA score (0.0475), followed by antihypertensive medication exposure (0.0421), weight (0.0323), age (0.0295), respiratory rate (0.0221), sepsis (0.0220), pH (0.0204), SpO2 (0.0189), platelet count (0.0151), diastolic blood pressure (0.0128), urine output (absolute coefficient 0.0119), magnesium (0.0102), systolic blood pressure (0.0100), temperature (0.0100), and heart rate (0.0053). Collectively, the retained variables represented early illness severity, baseline vulnerability, medication exposure, cardiopulmonary instability, acid-base status, hematologic response, and renal-fluid output signals.

### 3.3 Model performance in the MIMIC-IV test set

**Figure 3:**
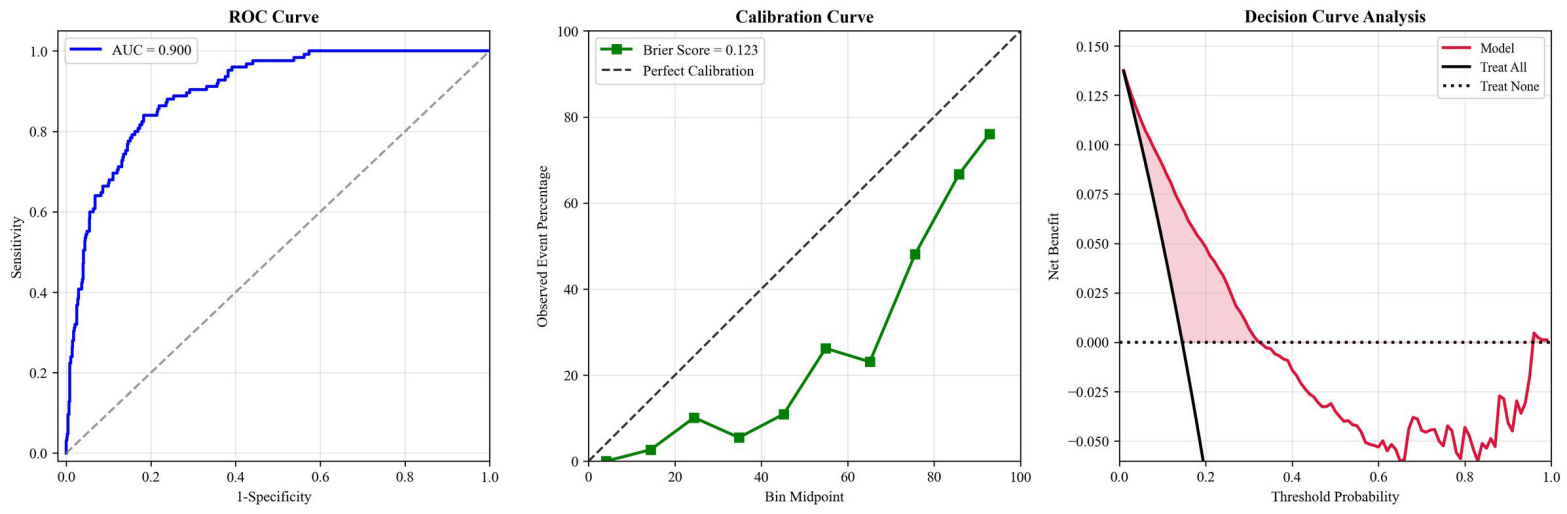
Logistic regression performance in the MIMIC-IV internal test set, including ROC curve, calibration curve, and decision curve analysis.

**Figure 4:**
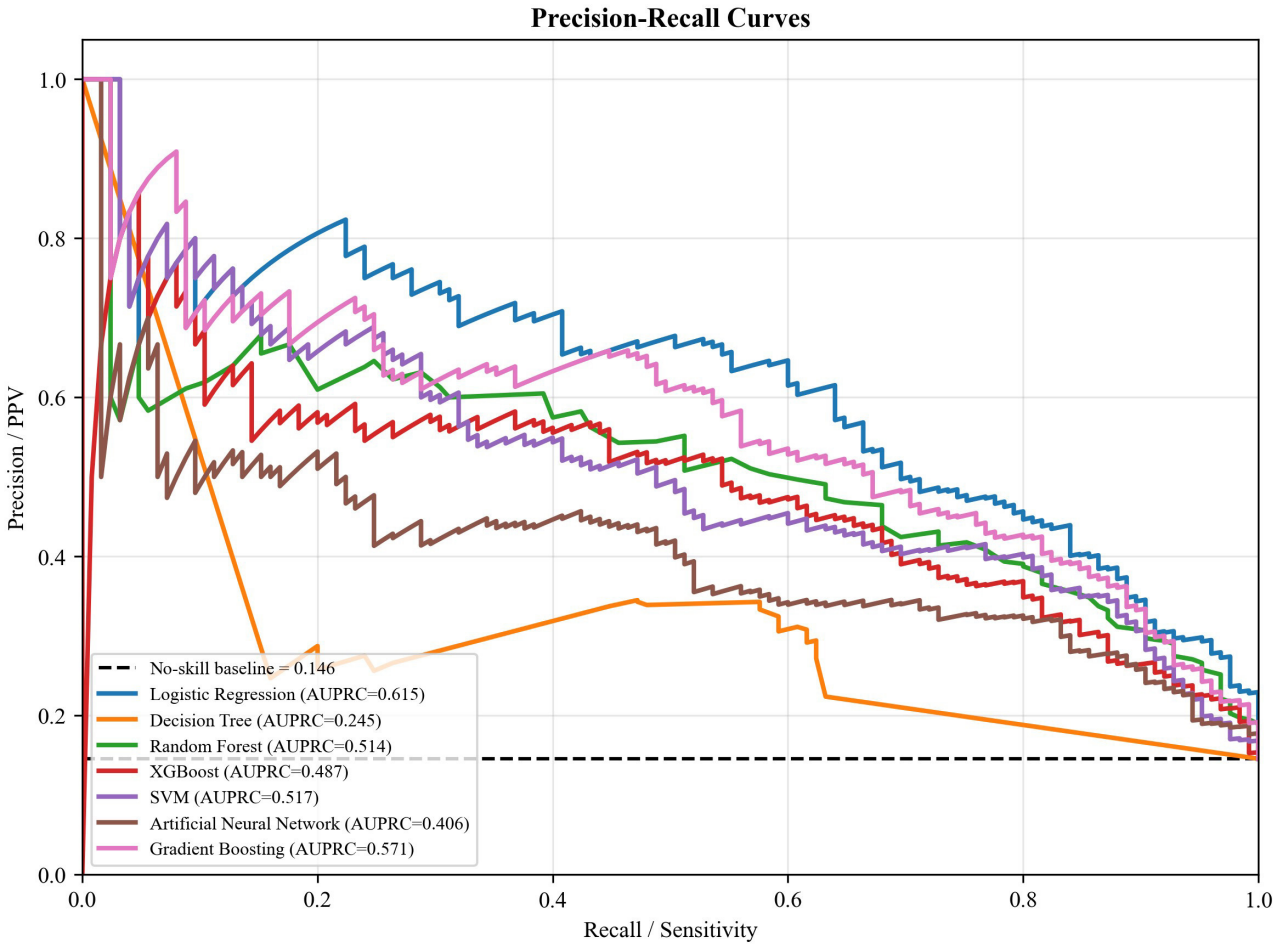
Precision-recall curves for all seven models in the MIMIC-IV internal test set. The no-skill baseline corresponds to AKI prevalence in the test set.

The comparative performance of the seven models in the MIMIC-IV internal test set is shown in Table 1, with the main performance plots provided in Figures 3, 4 and additional model-specific plots in Supplementary Figures S1–S6. Logistic regression achieved the highest ROC-AUC (0.900, 95% CI 0.878–0.923) and the highest AUPRC (0.615, 95% CI 0.533–0.703), exceeding the no-skill precision-recall baseline defined by AKI prevalence. At the training-set-derived cutoff of 0.363, logistic regression yielded sensitivity 0.888 (95% CI 0.837–0.935), specificity 0.716 (95% CI 0.685–0.750), positive predictive value 0.348 (95% CI 0.295–0.403), negative predictive value 0.974 (95% CI 0.961–0.986), F1 score 0.500, and Brier score 0.123. Gradient boosting and random forest also showed strong discrimination, with AUCs of 0.882 and 0.868, respectively. Gradient boosting achieved the highest F1 score (0.556) and the lowest Brier score (0.113), XGBoost achieved the highest specificity and positive predictive value, whereas random forest also showed relatively high specificity and positive predictive value. Given the combination of the highest ROC-AUC and AUPRC, high sensitivity and negative predictive value, favorable screening performance, and transparent implementation, logistic regression was selected as the primary model. Decision curve analysis supported positive net benefit across clinically relevant low-to-moderate threshold probabilities, indicating that the model is most appropriate as an early screening and monitoring aid rather than a definitive diagnostic tool.

**Table 1:** Internal validation performance of seven models in the MIMIC-IV test set.

Models	AUC (95%CI)	AUPRC (95%CI)	Cutoff	SEN (95%CI)	SPE (95%CI)	PPV (95%CI)	NPV (95%CI)	F1	Brier
Logistic regression	0.900 (0.878–0.923)	0.615 (0.533–0.703)	0.363	0.888 (0.837–0.935)	0.716 (0.685–0.750)	0.348 (0.295–0.403)	0.974 (0.961–0.986)	0.500	0.123
Decision tree	0.670 (0.618–0.720)	0.245 (0.196–0.299)	0.500	0.616 (0.524–0.703)	0.764 (0.730–0.796)	0.308 (0.253–0.366)	0.921 (0.894–0.942)	0.411	0.223
Random forest	0.868 (0.837–0.899)	0.514 (0.426–0.606)	0.570	0.552 (0.468–0.649)	0.914 (0.894–0.931)	0.523 (0.443–0.603)	0.923 (0.899–0.943)	0.537	0.115
XGBoost	0.847 (0.813–0.881)	0.487 (0.417–0.578)	0.795	0.448 (0.362–0.530)	0.940 (0.923–0.959)	0.560 (0.475–0.657)	0.909 (0.881–0.929)	0.498	0.123
Svm	0.852 (0.817–0.890)	0.517 (0.437–0.603)	0.330	0.824 (0.756–0.887)	0.760 (0.732–0.792)	0.369 (0.313–0.428)	0.962 (0.944–0.976)	0.510	0.131
Artificial neural network	0.812 (0.776–0.844)	0.406 (0.333–0.495)	0.523	0.576 (0.485–0.651)	0.823 (0.800–0.850)	0.356 (0.300–0.425)	0.919 (0.894–0.938)	0.440	0.159
Gradient boosting	0.882 (0.853–0.910)	0.571 (0.490–0.658)	0.503	0.712 (0.630–0.786)	0.855 (0.826–0.879)	0.456 (0.387–0.525)	0.946 (0.926–0.962)	0.556	0.113

AUC, area under the receiver operating characteristic curve; AUPRC, area under the precision-recall curve; SEN, sensitivity; SPE, specificity; PPV, positive predictive value; NPV, negative predictive value; CI, confidence interval.

### 3.4 External validation in the eICU-CRD cohort

The external validation cohort from eICU-CRD included 1,772 polytrauma patients, of whom 471 developed AKI after outcome recoding, corresponding to an event rate of 26.6%. Overall model performance in the external validation cohort was more modest than that observed in the MIMIC-IV internal test set, which may reflect differences in database structure, case mix, feature harmonization, clinical practice patterns, and outcome ascertainment between the two data sources (Table 2; Supplementary Figure S15). Among the evaluated models, SVM achieved the highest discrimination and precision-recall performance in the eICU-CRD cohort, with an AUC of 0.709 (95% CI 0.649–0.765) and an AUPRC of 0.480 (95% CI 0.374–0.576). At the selected cutoff, SVM showed a sensitivity of 0.617, specificity of 0.682, positive predictive value of 0.411, and negative predictive value of 0.832. The interpretable logistic regression model also demonstrated moderate external performance, with an AUC of 0.678 and an AUPRC of 0.466. These findings suggest that the selected predictor set retained partial transportability in an independent external database, while the attenuated performance highlights the importance of local recalibration and prospective multicenter validation before clinical deployment.

**Table 2:** External validation performance in the eICU-CRD cohort.

Model	AUC (95% CI)	AUPRC (95% CI)	SEN	SPE	PPV	NPV
SVM	0.709 (0.649–0.765)	0.480 (0.374–0.576)	0.617	0.682	0.411	0.832
Decision tree	0.692 (0.631–0.748)	0.461 (0.361–0.548)	0.681	0.628	0.398	0.845
Gradient boosting	0.683 (0.618–0.743)	0.447 (0.347–0.534)	0.564	0.663	0.376	0.808
Logistic regression	0.678 (0.612–0.734)	0.466 (0.365–0.558)	0.702	0.563	0.367	0.840
XGBoost	0.676 (0.611–0.733)	0.428 (0.337–0.511)	0.564	0.716	0.417	0.820
Random forest	0.676 (0.610–0.734)	0.443 (0.344–0.529)	0.553	0.709	0.406	0.815
Artificial neural network	0.668 (0.602–0.725)	0.443 (0.350–0.533)	0.479	0.747	0.405	0.799

The eICU-CRD analysis used the same retained predictor set. Abbreviations: AUC, area under the receiver operating characteristic curve; AUPRC, area under the precision-recall curve; SEN, sensitivity; SPE, specificity; PPV, positive predictive value; NPV, negative predictive value; CI, confidence interval.

### 3.5 SHAP interpretation of the primary model

**Figure 5:**
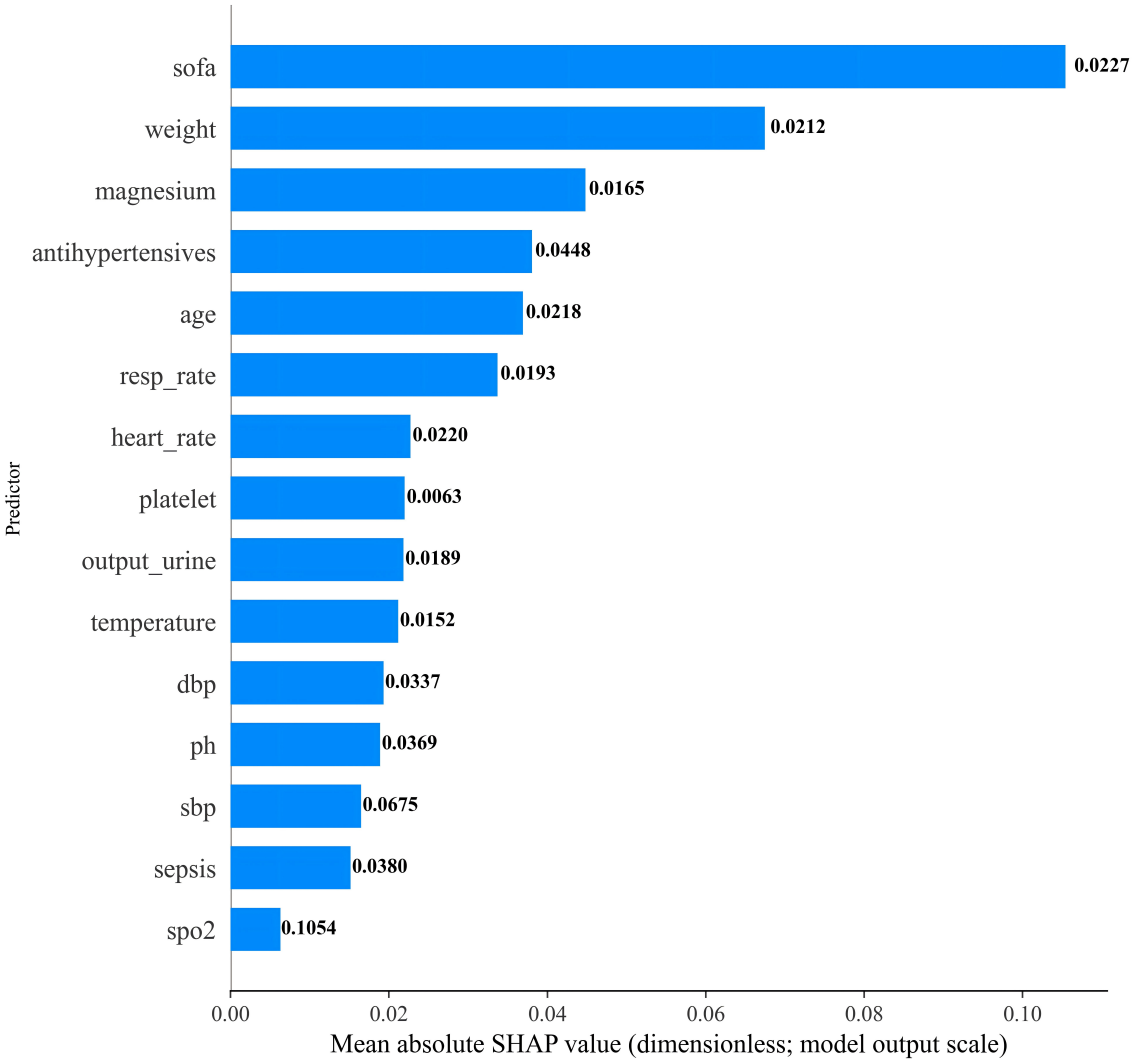
SHAP summary bar plot showing global feature importance of the primary logistic regression model.

**Figure 6:**
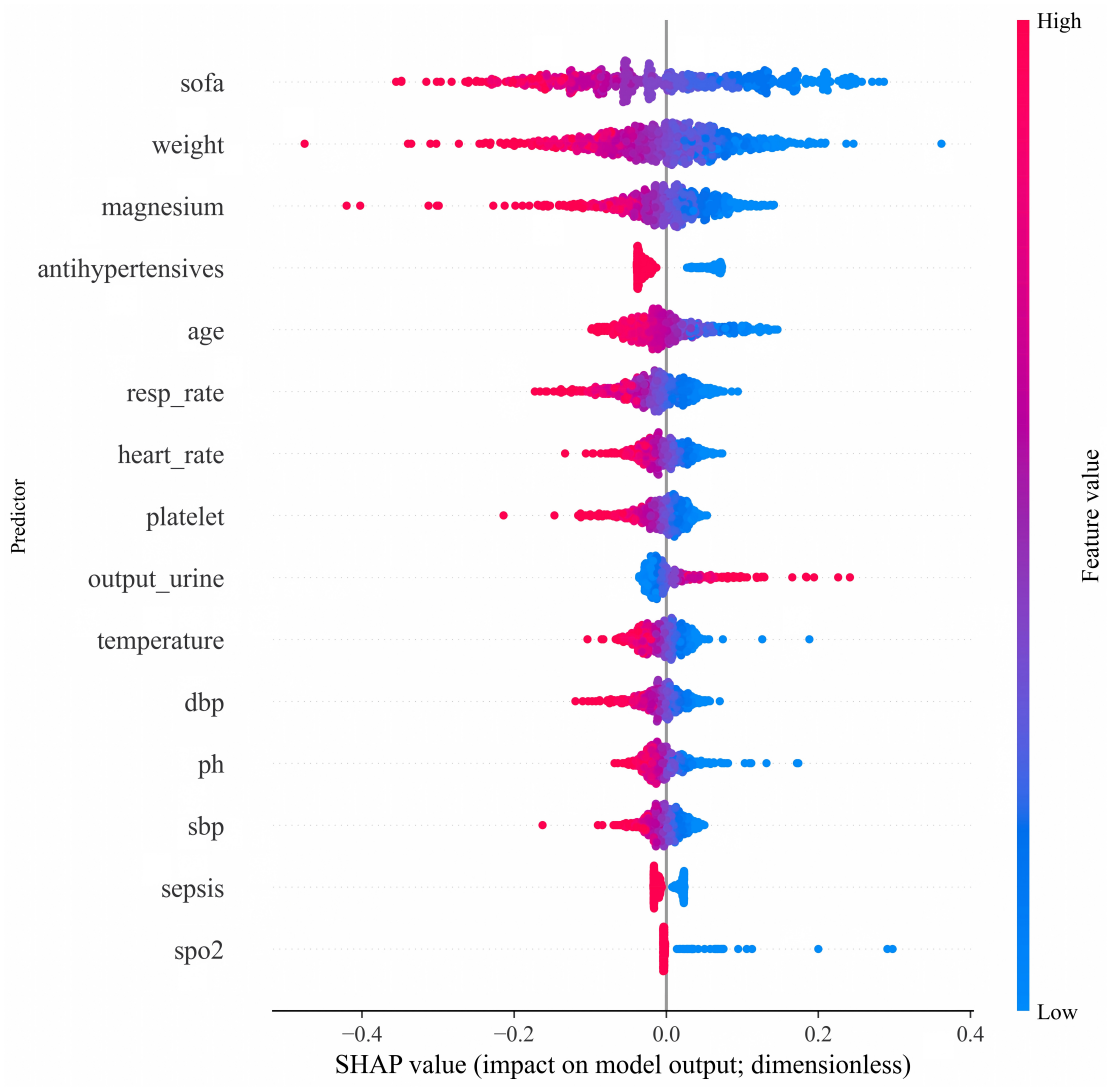
SHAP summary dot plot showing feature-level contributions to individual predictions.

**Figure 7:**
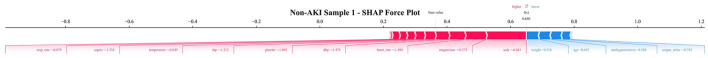
Representative SHAP force plot for a non-AKI case.

**Figure 8:**
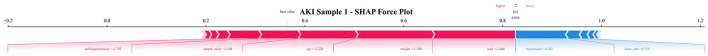
Representative SHAP force plot for an AKI case.

SHAP analysis was performed for the primary logistic regression model to examine global and individual-level model behavior. Mean absolute SHAP values ranked SOFA score, weight, magnesium, antihypertensive medication exposure, age, respiratory rate, heart rate, platelet count, urine output, and temperature among the most influential contributors (Figure 5). The SHAP summary plot demonstrated heterogeneous but clinically coherent feature effects across patients, indicating that the final prediction was shaped by the joint contribution of severity, cardiopulmonary, hematologic, renal-fluid, electrolyte, and baseline patient-level signals rather than by any single predictor alone (Figure 6). Individual force plots further illustrated how the same feature set produced distinct risk profiles in representative non-AKI and AKI cases (Figures 7, 8). Additional individual-level examples are provided in Supplementary Figures S7–S14.

## 4 Discussion

Using routinely collected data from the first 6 h after ICU admission, this study developed and interpreted an early AKI prediction framework for adult ICU patients with polytrauma. The main finding was that a compact 15-variable logistic regression model achieved the best overall internal discrimination and precision-recall performance in the MIMIC-IV test set, with an AUC of 0.900 and an AUPRC of 0.615. At the training-set-derived cutoff, the model showed high sensitivity and a high negative predictive value, supporting its role as an early screening aid rather than a definitive diagnostic tool. In the secondary eICU-CRD analysis, model performance was attenuated but remained informative, indicating partial cross-database transportability of the selected predictor set.

The retained predictors were clinically plausible and reflected the multifactorial pathophysiology of trauma-associated AKI. Polytrauma can induce kidney injury through overlapping mechanisms, including hemorrhagic shock, systemic inflammation, ischemia-reperfusion injury, endothelial dysfunction, microcirculatory disturbance, rhabdomyolysis, and exposure to nephrotoxic or hemodynamically active treatments (9). The strong contribution of SOFA score is therefore expected, because this score summarizes early multisystem organ dysfunction and captures clinically relevant derangements across respiratory, circulatory, coagulation, hepatic, neurologic, and renal domains (24). Sepsis was also retained by LASSO, supporting the biological link between dysregulated host response, organ dysfunction, and secondary kidney injury in critically ill trauma patients (25). Age and weight further suggest that baseline vulnerability, physiologic reserve, and treatment complexity may modify AKI susceptibility after major injury.

Several dynamic physiologic variables selected by the model are also consistent with early kidney-risk pathways. Respiratory rate, heart rate, systolic and diastolic blood pressure, SpO_2_, and temperature may reflect cardiopulmonary instability, inflammatory burden, shock physiology, and resuscitation status. These signals are particularly relevant after trauma, where renal perfusion and oxygen delivery can change rapidly during the early ICU period. Platelet count may represent coagulation disturbance and inflammatory-endothelial activation, whereas urine output provides an early functional signal of renal-fluid homeostasis. Although urine output is part of conventional AKI diagnosis, its use within the first 6-h predictor window and the subsequent 7–72-h outcome window maintains temporal separation and supports its value as an early warning indicator rather than a delayed diagnostic marker.

Magnesium and antihypertensive medication exposure require more cautious interpretation. Serum magnesium may represent electrolyte imbalance, renal handling, tissue injury, or treatment-related variation in critical illness, rather than a direct causal driver of AKI. Similarly, antihypertensive medication exposure may indicate underlying cardiovascular comorbidity, chronic vascular vulnerability, or hemodynamic susceptibility. Because SHAP quantifies feature contributions to model predictions rather than causal effects, these findings should be interpreted as clinically meaningful risk-stratification signals rather than mechanistic proof (26). This distinction is important when translating explainable machine-learning outputs into bedside decision-making.

The finding that logistic regression outperformed more complex models in internal discrimination and precision-recall performance is notable but clinically reasonable. Previous AKI prediction studies have often reported strong performance for tree-based or boosting algorithms, particularly in heterogeneous ICU cohorts and continuous prediction settings (27). However, in trauma-specific prediction tasks, simpler models may remain competitive when the prediction window is clearly defined, the feature set is parsimonious, and clinically strong early signals are available. Recent trauma-focused studies also suggest that machine-learning algorithms do not always provide a decisive advantage over well-specified regression models when interpretability, calibration, and deployment feasibility are considered (28). In the present study, LASSO feature reduction may have limited the incremental benefit of highly flexible nonlinear models, while logistic regression retained advantages in transparency, stability, and ease of clinical implementation.

The eICU-CRD validation results provide an important cautionary message. Although SVM achieved the highest external AUC and AUPRC, all models performed less well externally than internally. This decrease is expected in cross-database validation because differences in hospital composition, case mix, coding systems, injury spectrum, laboratory measurement patterns, urine output capture, treatment workflows, and AKI ascertainment can substantially affect model transportability. Prediction-model reviews have repeatedly shown that AKI models often demonstrate reduced performance when externally validated in new populations or institutions (29). Therefore, the eICU-CRD findings should not be interpreted as model failure. Rather, they indicate that the selected predictor set contains transferable information but requires local recalibration before routine use.

From a clinical perspective, the primary value of this model lies in early risk enrichment. The high sensitivity and negative predictive value of logistic regression suggest that it may help identify patients who warrant closer kidney monitoring, repeated assessment of serum creatinine and urine output, medication review, hemodynamic optimization, and avoidance of potentially nephrotoxic exposure. Decision curve analysis further supports its potential usefulness across low-to-moderate threshold probabilities, which is consistent with a screening-oriented application. Such a model should not replace KDIGO-based diagnosis, clinician judgment, or nephrology consultation. Instead, it may serve as an early warning layer within a kidney-protective care pathway, prompting individualized review before overt AKI criteria are met.

This study has several strengths. First, the predictor window and outcome window were explicitly separated: predictors were extracted from the first 6 h after ICU admission, whereas incident AKI was assessed between 7 and 72 h. Second, feature selection, imputation, standardization, resampling, hyperparameter tuning, and threshold selection were restricted to the training set, reducing the risk of information leakage. Third, model evaluation included ROC-AUC, AUPRC, threshold-based classification metrics, calibration, Brier score, decision curve analysis, and SHAP interpretation. This broader evaluation strategy is consistent with current recommendations that prediction models should be assessed beyond discrimination alone (30, 31). Fourth, the final model used only 15 routinely available variables, which improves clinical interpretability and workflow feasibility.

Several limitations should be acknowledged. First, this was a retrospective study based on public critical care databases, so residual confounding, coding errors, missingness, and unmeasured clinical factors cannot be fully excluded. Second, polytrauma and AKI were operationalized using structured database variables; although harmonized definitions were applied, misclassification may still have occurred. Third, classical trauma severity scores, such as the Injury Severity Score (ISS) and the Revised Trauma Score (RTS), were not incorporated into the current model because they were not directly recorded in MIMIC-IV or eICU-CRD and could not be reliably reconstructed from the available database variables. This may limit trauma-severity adjustment and comparability with studies based on dedicated trauma registries. Fourth, baseline creatinine was estimated using a surrogate value when preadmission creatinine was unavailable, which is common in database studies but may influence AKI classification. Fifth, external validation showed only moderate transportability, emphasizing the need for prospective multicenter validation and local recalibration in contemporary trauma ICU settings. Sixth, the model did not incorporate kidney injury biomarkers, high-frequency physiologic waveforms, or longitudinal resuscitation trajectories, which may further improve early prediction and risk phenotyping in future work (32–34).

Future research should therefore focus on prospective validation, local model updating, and implementation-impact assessment. Before clinical deployment, the model should be tested across diverse trauma centers to evaluate calibration, threshold stability, and workflow compatibility. Future prospective studies or trauma-registry-based studies should also incorporate classical trauma severity scores, including ISS and RTS, to further improve trauma-severity adjustment and model generalizability. Subsequent implementation studies should determine whether model-guided early warning improves kidney-protective bundle adherence, nephrotoxin review, early nephrology consultation, AKI severity, need for kidney replacement therapy, ICU length of stay, and mortality.

## 5 Conclusion

A 15-variable, interpretable logistic regression model using data from the first 6 h after ICU admission achieved strong internal discrimination and precision-recall performance for early AKI prediction in polytrauma patients. The model provides clinically readable risk signals and may support timely kidney-protective monitoring, while the secondary eICU-CRD analysis indicates that cross-database transportability is moderate and should be strengthened through prospective multicenter validation and local recalibration.

## Data Availability

Publicly available datasets were analyzed in this study. The MIMIC-IV database can be found at https://physionet.org/content/mimiciv/, and the eICU Collaborative Research Database can be found at https://physionet.org/content/eicu-crd/.
