# Supplementary material for: An interpretable machine-learning model for early prediction of acute kidney injury in polytrauma patients
Source: Front Med (Lausanne). 2026 Jul 20;13:1750815. doi: 10.3389/fmed.2026.1750815 (PMC13429611; doi:10.3389/fmed.2026.1750815)

**Supplementary Material**

**An Interpretable Machine-Learning Model for Early Prediction of Acute Kidney Injury in Polytrauma Patients**

# **Supplementary Tables**

**Supplementary Table S1.** Baseline characteristics of the MIMIC-IV primary cohort.

| **Characteristic** | **Category** | **Total** | **AKI (n=623)** | **Non-AKI (n=3664)** | **P-value** |
| --- | --- | --- | --- | --- | --- |
| gender | 0 | 1626 (37.9) | 251 (40.3) | 1375 (37.5) | 0.205 |
|  | 1 | 2661 (62.1) | 372 (59.7) | 2289 (62.5) |  |
| smoker | 0 | 4139 (96.5) | 597 (95.8) | 3542 (96.7) | 0.343 |
|  | 1 | 148 (3.5) | 26 (4.2) | 122 (3.3) |  |
| alcohol_abuse | 0 | 4260 (99.4) | 622 (99.8) | 3638 (99.3) | 0.184 |
|  | 1 | 27 (0.6) | 1 (0.2) | 26 (0.7) |  |
| hypertension | 0 | 2700 (63.0) | 409 (65.7) | 2291 (62.5) | 0.148 |
|  | 1 | 1587 (37.0) | 214 (34.3) | 1373 (37.5) |  |
| diabetes | 1 | 1161 (27.1) | 128 (20.5) | 1033 (28.2) | <0.001 |
|  | 0 | 3126 (72.9) | 495 (79.5) | 2631 (71.8) |  |
| myocardial_infarct | 1 | 881 (20.6) | 83 (13.3) | 798 (21.8) | <0.001 |
|  | 0 | 3406 (79.4) | 540 (86.7) | 2866 (78.2) |  |
| congestive_heart_failure | 0 | 3120 (72.8) | 530 (85.1) | 2590 (70.7) | <0.001 |
|  | 1 | 1167 (27.2) | 93 (14.9) | 1074 (29.3) |  |
| renal_disease | 1 | 764 (17.8) | 67 (10.8) | 697 (19.0) | <0.001 |
|  | 0 | 3523 (82.2) | 556 (89.2) | 2967 (81.0) |  |
| cerebrovascular_disease | 0 | 3563 (83.1) | 552 (88.6) | 3011 (82.2) | <0.001 |
|  | 1 | 724 (16.9) | 71 (11.4) | 653 (17.8) |  |
| sirs | 1 | 335 (7.8) | 52 (8.3) | 283 (7.7) | 0.008 |
|  | 3 | 1905 (44.4) | 277 (44.5) | 1628 (44.4) |  |
|  | 4 | 855 (19.9) | 100 (16.1) | 755 (20.6) |  |
|  | 2 | 1163 (27.1) | 185 (29.7) | 978 (26.7) |  |
|  | 0 | 29 (0.7) | 9 (1.4) | 20 (0.5) |  |
| sepsis | 0 | 1530 (35.7) | 415 (66.6) | 1115 (30.4) | <0.001 |
|  | 1 | 2757 (64.3) | 208 (33.4) | 2549 (69.6) |  |
| InvasiveVent | 0 | 1183 (27.6) | 303 (48.6) | 880 (24.0) | <0.001 |
|  | 1 | 3104 (72.4) | 320 (51.4) | 2784 (76.0) |  |
| immunosuppressants | 0 | 4152 (96.9) | 619 (99.4) | 3533 (96.4) | <0.001 |
|  | 1 | 135 (3.1) | 4 (0.6) | 131 (3.6) |  |
| glucocorticoids | 0 | 3203 (74.7) | 515 (82.7) | 2688 (73.4) | <0.001 |
|  | 1 | 1084 (25.3) | 108 (17.3) | 976 (26.6) |  |
| antihypertensives | 1 | 3256 (76.0) | 317 (50.9) | 2939 (80.2) | <0.001 |
|  | 0 | 1031 (24.0) | 306 (49.1) | 725 (19.8) |  |
| cam_icu | 0 | 3450 (80.5) | 582 (93.4) | 2868 (78.3) | <0.001 |
|  | 1 | 837 (19.5) | 41 (6.6) | 796 (21.7) |  |
| age, median (IQR) |  | 65.68 (53.32-75.76) | 61.39 (44.50-73.30) | 66.40 (54.36-76.08) | <0.001 |
| height, median (IQR) |  | 170.00 (163.00-178.00) | 170.00 (163.00-178.00) | 170.00 (163.00-178.00) | 0.099 |
| weight, median (IQR) |  | 80.00 (68.00-96.60) | 74.00 (63.00-86.25) | 81.60 (69.00-98.00) | <0.001 |
| sofa, median (IQR) |  | 7.00 (4.00-10.00) | 4.00 (2.00-6.00) | 7.00 (5.00-10.00) | <0.001 |
| gcs, median (IQR) |  | 13.00 (8.00-15.00) | 14.00 (13.00-15.00) | 13.00 (7.00-14.00) | <0.001 |
| output_urine, median (IQR) |  | 150.00 (75.00-300.00) | 200.00 (100.00-375.00) | 150.00 (70.00-300.00) | <0.001 |
| temperature, median (IQR) |  | 37.89 (37.33-38.78) | 37.40 (37.11-37.94) | 38.00 (37.39-38.90) | <0.001 |
| heart_rate, median (IQR) |  | 121.00 (105.00-138.00) | 109.00 (96.00-126.00) | 123.00 (107.00-140.00) | <0.001 |
| resp_rate, median (IQR) |  | 35.00 (29.00-41.00) | 29.00 (25.00-35.00) | 36.00 (30.00-42.00) | <0.001 |
| sbp, median (IQR) |  | 165.00 (148.00-184.00) | 152.00 (137.00-169.00) | 168.00 (150.00-186.00) | <0.001 |
| dbp, median (IQR) |  | 105.00 (90.00-121.00) | 93.00 (82.00-107.50) | 107.00 (92.00-123.00) | <0.001 |
| mbp, median (IQR) |  | 122.00 (106.00-146.00) | 110.00 (98.00-125.00) | 124.00 (109.00-149.00) | <0.001 |
| spo2, median (IQR) |  | 100.00 (100.00-100.00) | 100.00 (100.00-100.00) | 100.00 (100.00-100.00) | <0.001 |
| ph, median (IQR) |  | 7.45 (7.40-7.49) | 7.41 (7.37-7.45) | 7.46 (7.41-7.50) | <0.001 |
| pao2fio2ratio, median (IQR) |  | 357.50 (258.00-462.50) | 335.00 (225.71-471.00) | 359.29 (264.75-462.00) | 0.002 |
| po2, median (IQR) |  | 203.00 (133.50-329.00) | 197.00 (111.00-322.00) | 205.00 (137.00-330.00) | 0.002 |
| pco2, median (IQR) |  | 47.00 (41.00-56.00) | 44.00 (37.50-50.00) | 48.00 (41.00-57.00) | <0.001 |
| lactate, median (IQR) |  | 2.30 (1.50-4.00) | 2.00 (1.30-3.20) | 2.40 (1.50-4.20) | <0.001 |
| wbc, median (IQR) |  | 17.70 (13.40-23.80) | 14.50 (11.60-19.25) | 18.20 (13.90-24.70) | <0.001 |
| lymphocytes_abs, median (IQR) |  | 1.54 (1.00-2.27) | 1.50 (0.92-2.25) | 1.54 (1.01-2.27) | 0.327 |
| neutrophils_abs, median (IQR) |  | 11.24 (7.88-16.64) | 9.81 (6.63-13.39) | 11.57 (8.08-17.25) | <0.001 |
| monocytes_abs, median (IQR) |  | 0.95 (0.58-1.43) | 0.76 (0.47-1.12) | 0.99 (0.62-1.48) | <0.001 |
| platelet, median (IQR) |  | 306.00 (217.00-439.00) | 262.00 (197.00-346.50) | 317.00 (220.00-454.00) | <0.001 |
| rbc, median (IQR) |  | 4.00 (3.52-4.50) | 4.04 (3.59-4.55) | 3.99 (3.52-4.50) | 0.154 |
| albumin, median (IQR) |  | 3.40 (2.90-3.80) | 3.50 (3.00-3.90) | 3.30 (2.90-3.80) | <0.001 |
| creatinine, median (IQR) |  | 1.30 (0.90-2.30) | 1.00 (0.80-1.30) | 1.40 (1.00-2.60) | <0.001 |
| bun, median (IQR) |  | 32.00 (21.00-56.00) | 19.00 (14.00-27.00) | 35.00 (23.00-61.00) | <0.001 |
| aniongap, median (IQR) |  | 18.00 (15.00-21.00) | 16.00 (14.00-19.00) | 18.00 (16.00-22.00) | <0.001 |
| calcium_total, median (IQR) |  | 9.20 (8.70-9.70) | 9.00 (8.60-9.40) | 9.20 (8.80-9.80) | <0.001 |
| pt, median (IQR) |  | 16.10 (13.70-22.60) | 14.30 (12.50-16.95) | 16.60 (14.00-23.90) | <0.001 |
| ptt, median (IQR) |  | 39.70 (31.00-94.55) | 31.90 (28.10-46.40) | 43.00 (31.70-103.12) | <0.001 |
| inr, median (IQR) |  | 1.50 (1.30-2.10) | 1.30 (1.20-1.60) | 1.50 (1.30-2.20) | <0.001 |
| alt, median (IQR) |  | 44.00 (22.00-127.00) | 29.00 (17.00-69.00) | 48.00 (23.00-140.00) | <0.001 |
| ast, median (IQR) |  | 60.00 (30.00-173.00) | 39.00 (24.00-86.00) | 66.00 (32.00-193.25) | <0.001 |
| bilirubin_total, median (IQR) |  | 0.80 (0.50-1.50) | 0.60 (0.40-0.90) | 0.80 (0.50-1.60) | <0.001 |
| chloride, median (IQR) |  | 110.00 (106.00-114.00) | 108.00 (105.00-111.00) | 110.00 (107.00-114.00) | <0.001 |
| fasting_blood_glucose, median (IQR) |  | 158.00 (129.00-215.00) | 132.00 (112.00-172.00) | 164.00 (133.00-223.00) | <0.001 |
| potassium, median (IQR) |  | 5.10 (4.60-5.70) | 4.70 (4.30-5.20) | 5.10 (4.70-5.70) | <0.001 |
| sodium, median (IQR) |  | 144.00 (141.00-149.00) | 142.00 (140.00-145.00) | 144.00 (141.00-150.00) | <0.001 |
| bicarbonate, median (IQR) |  | 29.00 (26.00-31.00) | 27.00 (25.00-29.00) | 29.00 (26.00-32.00) | <0.001 |
| hemoglobin, median (IQR) |  | 12.10 (10.70-13.60) | 12.20 (11.00-13.60) | 12.10 (10.60-13.60) | 0.040 |
| magnesium, median (IQR) |  | 2.60 (2.30-2.90) | 2.30 (2.10-2.60) | 2.60 (2.40-2.90) | <0.001 |
| phosphate, median (IQR) |  | 4.80 (4.10-6.20) | 4.20 (3.60-4.80) | 5.00 (4.20-6.50) | <0.001 |

Data are presented as n (%) for categorical variables and median (interquartile range) for continuous variables unless otherwise indicated. Category values retain the original database/modeling codes.

**Supplementary Table S2.** LASSO-selected predictors used for model development.

| **Feature** | **Coefficient** | **Absolute coefficient** |
| --- | --- | --- |
| sofa | 0.0475 | 0.0475 |
| antihypertensives | 0.0421 | 0.0421 |
| weight | 0.0323 | 0.0323 |
| age | 0.0295 | 0.0295 |
| resp_rate | 0.0221 | 0.0221 |
| sepsis | 0.0220 | 0.0220 |
| ph | 0.0204 | 0.0204 |
| spo2 | 0.0189 | 0.0189 |
| platelet | 0.0151 | 0.0151 |
| dbp | 0.0128 | 0.0128 |
| output_urine | -0.0119 | 0.0119 |
| magnesium | 0.0102 | 0.0102 |
| sbp | 0.0100 | 0.0100 |
| temperature | 0.0100 | 0.0100 |
| heart_rate | 0.0053 | 0.0053 |

Predictors are ranked by absolute LASSO coefficient.

**Supplementary Table S3.** AUPRC results for the seven models in the MIMIC-IV test set.

| **Models** | **AUPRC (95%CI)** |
| --- | --- |
| Logistic Regression | 0.615 (0.533–0.703) |
| Decision Tree | 0.245 (0.196–0.299) |
| Random Forest | 0.514 (0.426–0.606) |
| XGBoost | 0.487 (0.417–0.578) |
| SVM | 0.517 (0.437–0.603) |
| Artificial Neural Network | 0.406 (0.333–0.495) |
| Gradient Boosting | 0.571 (0.490–0.658) |

AUPRC, area under the precision-recall curve; CI, confidence interval.

# **Supplementary Figures**

**Supplementary Figure S1.** Artificial neural network evaluation in the MIMIC-IV internal test set: ROC curve, calibration curve, and decision curve analysis.


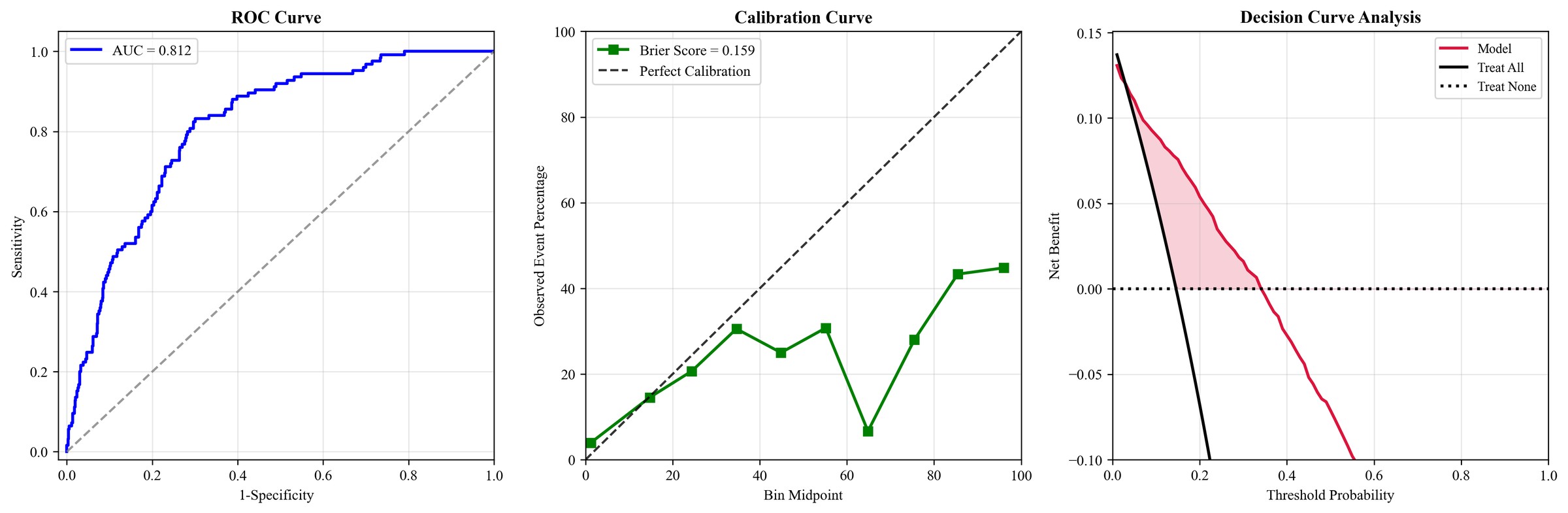


**Supplementary Figure S2.** Decision tree evaluation in the MIMIC-IV internal test set: ROC curve, calibration curve, and decision curve analysis.


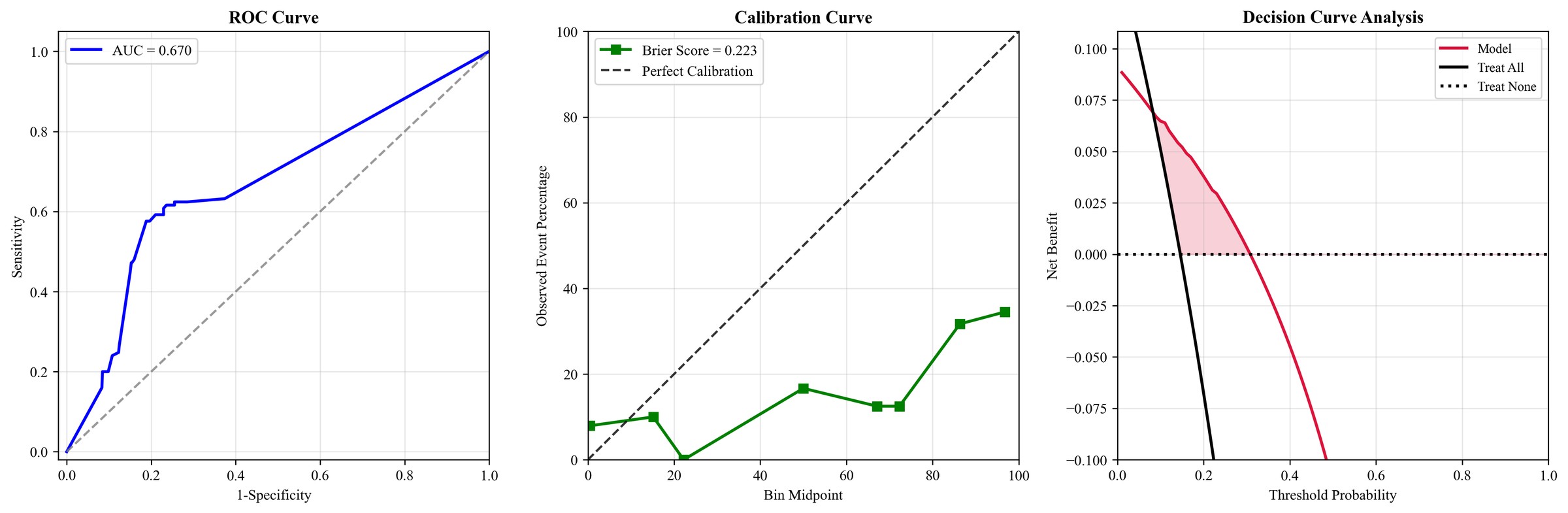


**Supplementary Figure S3.** Gradient boosting evaluation in the MIMIC-IV internal test set: ROC curve, calibration curve, and decision curve analysis.


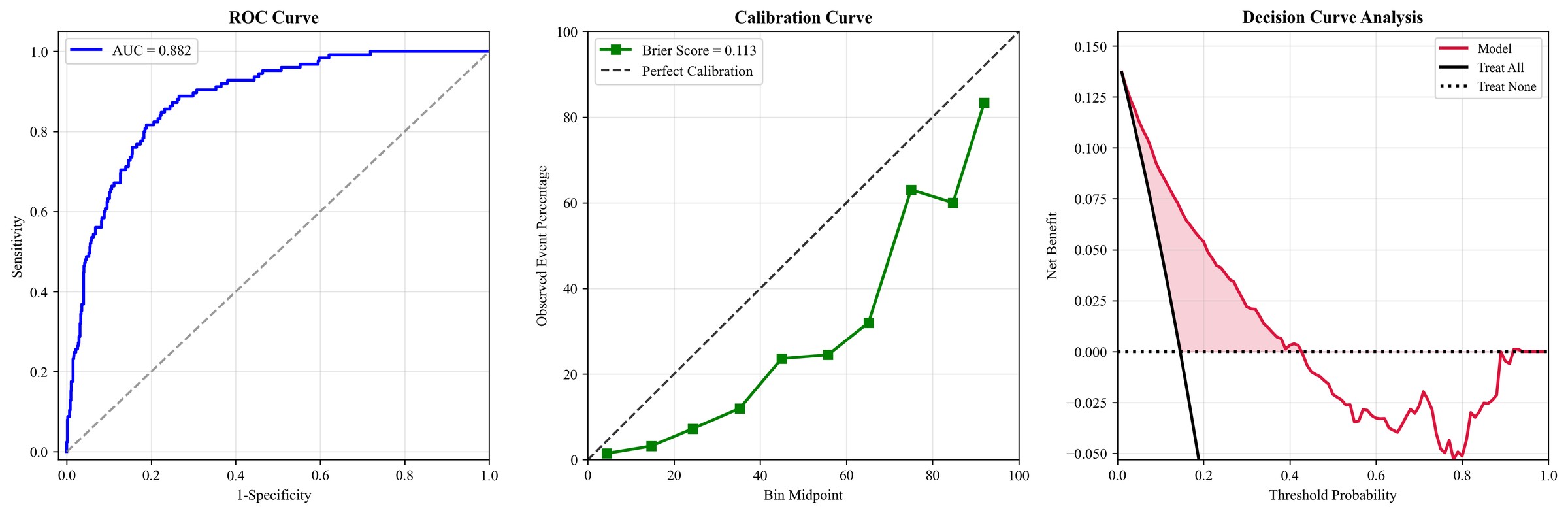


**Supplementary Figure S4.** Random forest evaluation in the MIMIC-IV internal test set: ROC curve, calibration curve, and decision curve analysis.


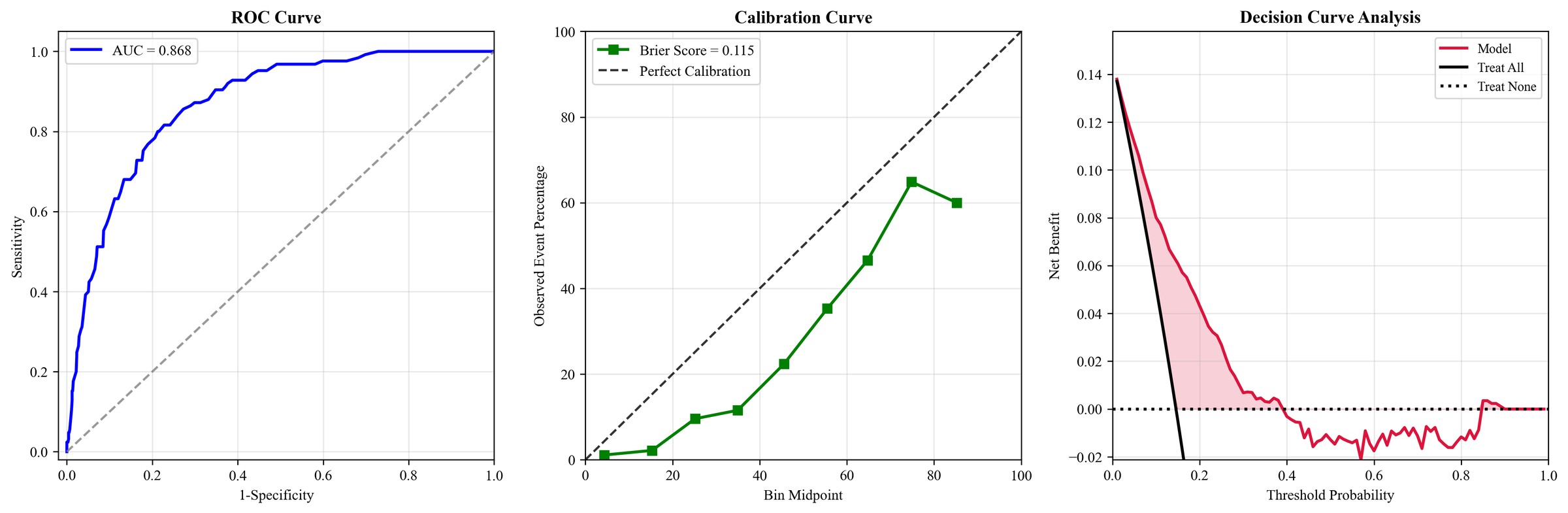


**Supplementary Figure S5.** Support vector machine evaluation in the MIMIC-IV internal test set: ROC curve, calibration curve, and decision curve analysis.


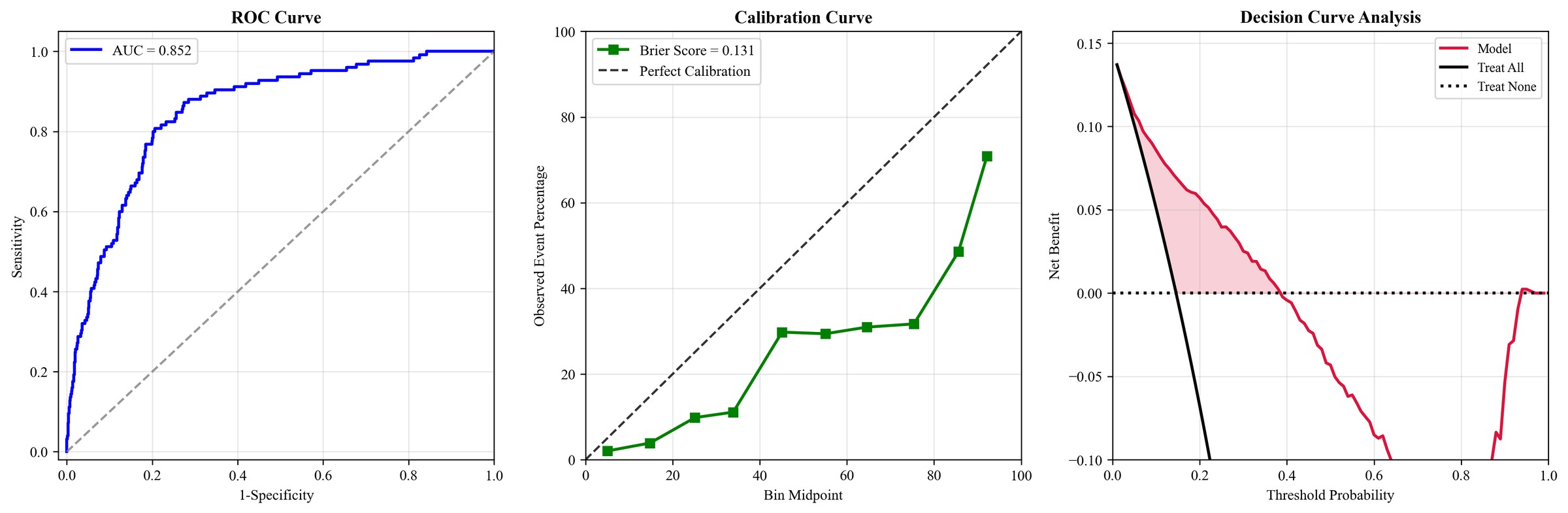


**Supplementary Figure S6.** XGBoost evaluation in the MIMIC-IV internal test set: ROC curve, calibration curve, and decision curve analysis.


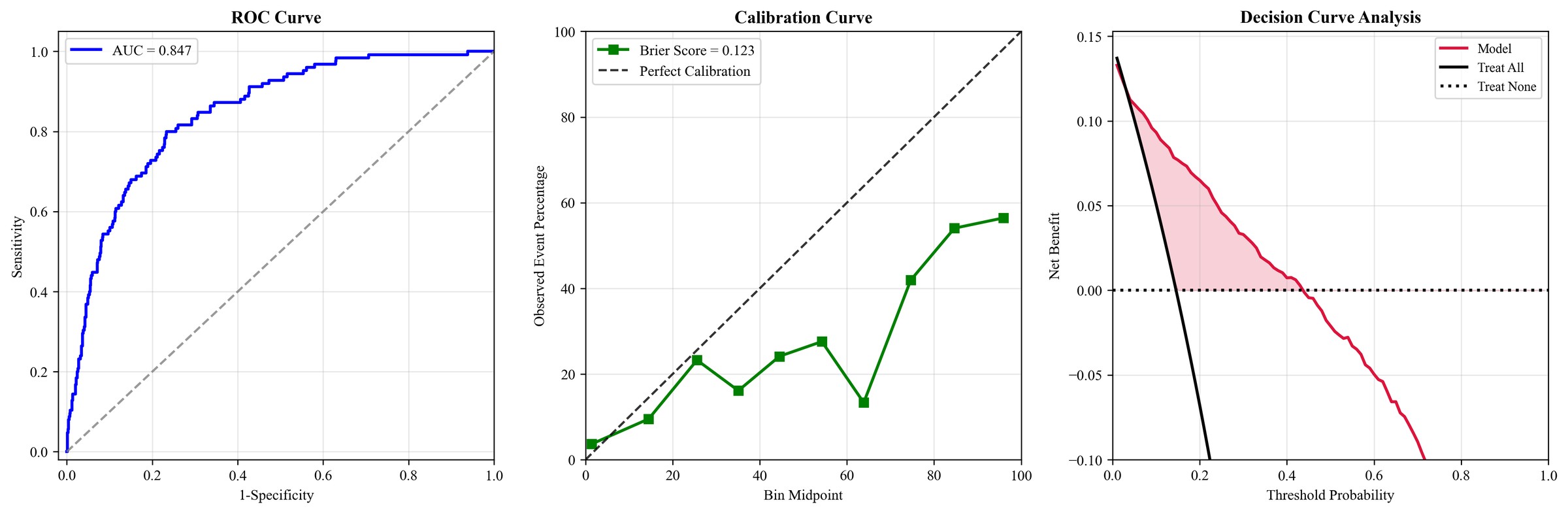


**Supplementary Figure S7.** SHAP force plot for additional AKI case 2.


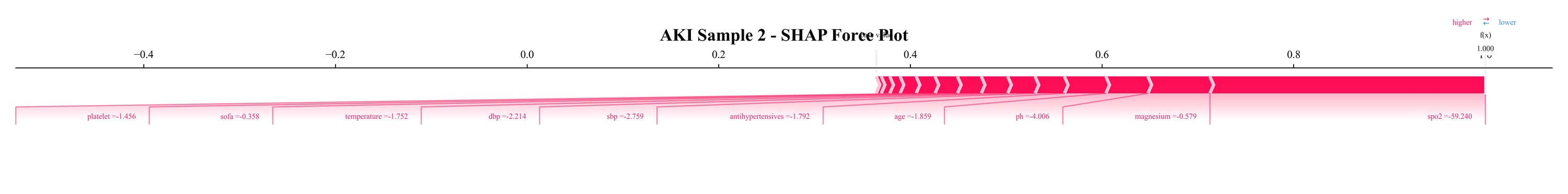


**Supplementary Figure S8.** SHAP force plot for additional AKI case 3.


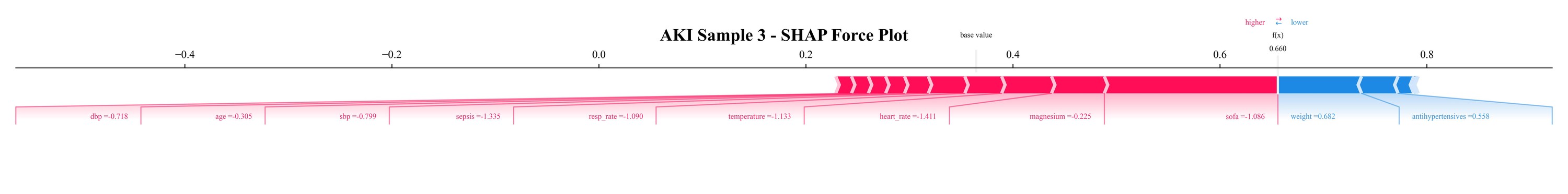


**Supplementary Figure S9.** SHAP force plot for additional AKI case 4.


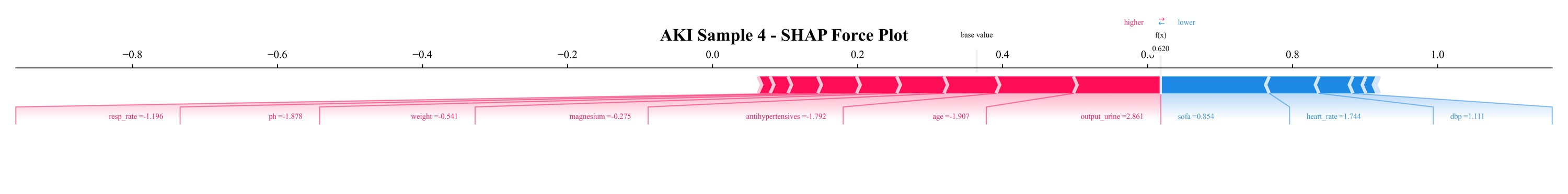


**Supplementary Figure S10.** SHAP force plot for additional AKI case 5.


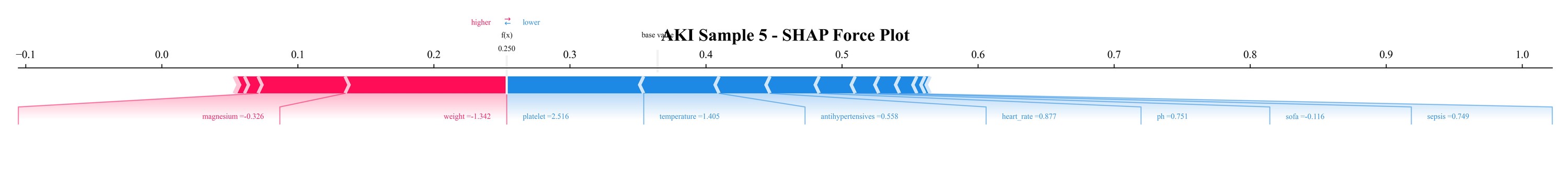


**Supplementary Figure S11.** SHAP force plot for additional non-AKI case 2.


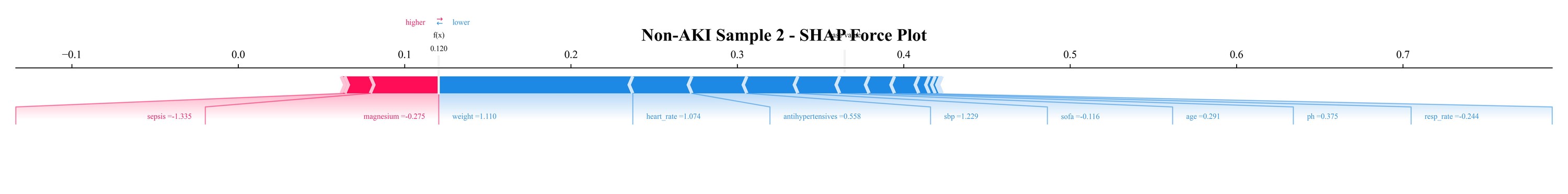


**Supplementary Figure S12.** SHAP force plot for additional non-AKI case 3.


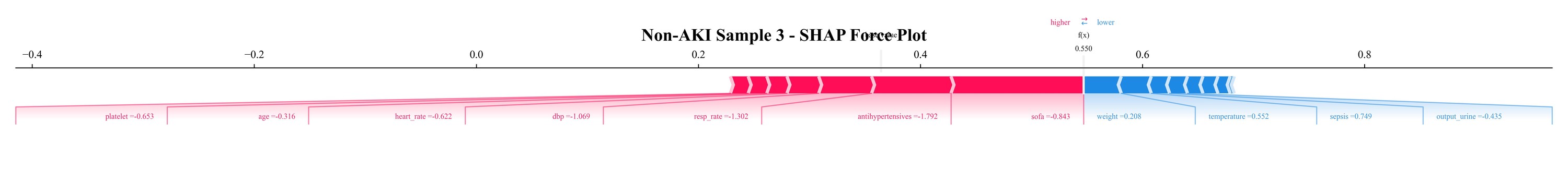


**Supplementary Figure S13.** SHAP force plot for additional non-AKI case 4.


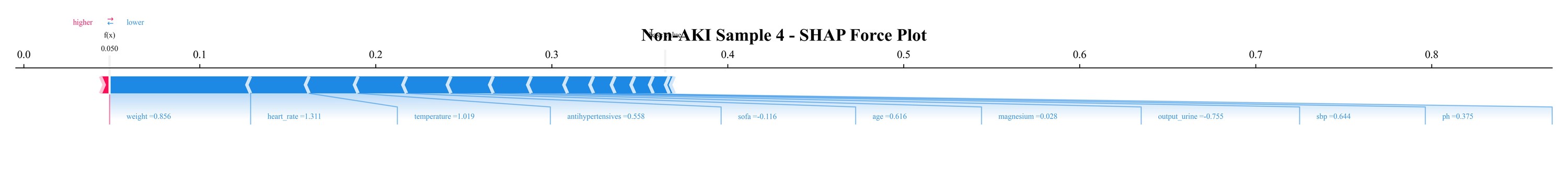


**Supplementary Figure S14.** SHAP force plot for additional non-AKI case 5.


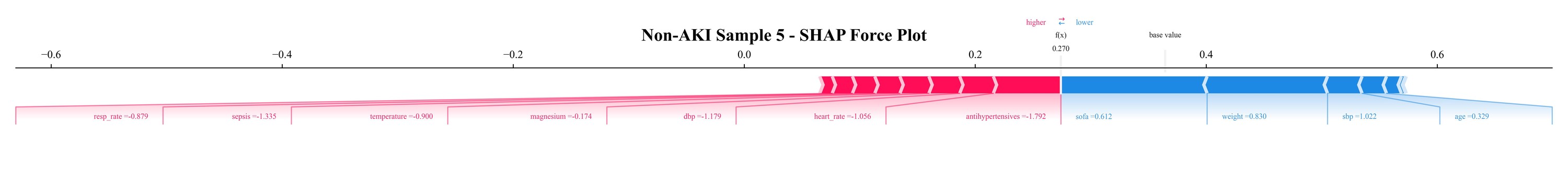


**Supplementary Figure S15.** Secondary eICU-CRD logistic regression performance plot, including ROC curve, precision-recall curve, decision curve analysis, and calibration curve.


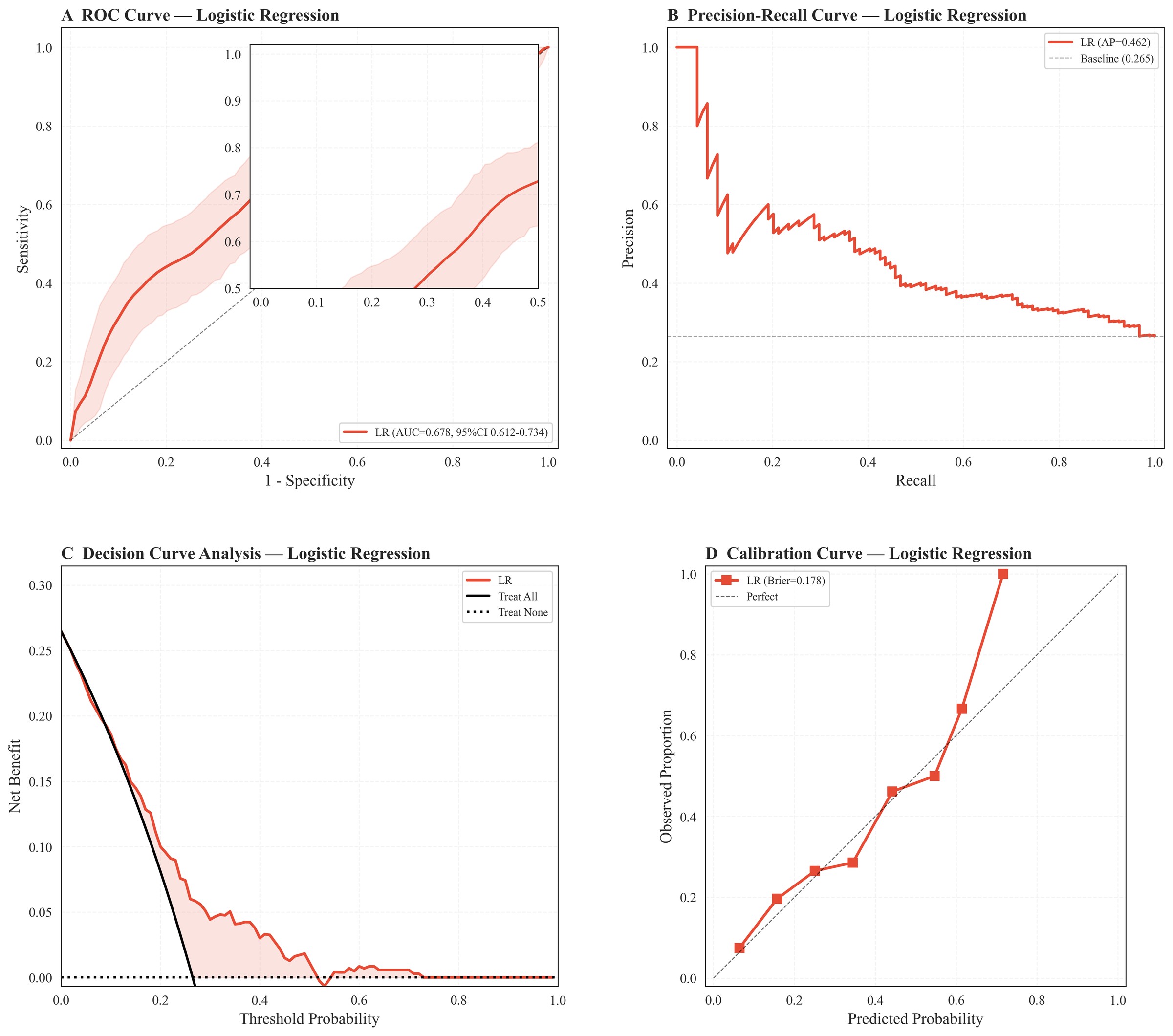

Supplement: Supplementary file 1 [file Table_1.docx]
